# Culture, acculturation and smoking use in Hmong, Khmer, Laotians, and Vietnamese communities in Minnesota

**DOI:** 10.1186/1471-2458-14-791

**Published:** 2014-08-04

**Authors:** Diana J Burgess, Jeremiah Mock, Barbara A Schillo, Jessie E Saul, Tam Phan, Yanat Chhith, Nina Alesci, Steven S Foldes

**Affiliations:** Minneapolis Veterans Affairs Healthcare System, Center for Chronic Disease Outcomes Research (CCDOR), One Veterans Drive, 152/2E, Minneapolis, MN 55417 USA; Department of Medicine, University of Minnesota, Minneapolis, MN USA; Center for the Study of Communication-Design, Osaka University, Suita, Japan; ClearWay Minnesota, Minneapolis, MN USA; North American Research & Analysis, Inc, Faribault, MN USA; Asian American Health Forum, San Francisco, CA USA; Southeast Asian Refugee Community Home, Minneapolis, MN USA; Blue Cross and Blue Shield of Minnesota, Eagan, MN USA; Foldes Consulting LLC, and Division of Epidemiology and Community Health, School of Public Health, University of Minnesota, Minneapolis, MN USA

**Keywords:** Tobacco, Smoking, Culture, Acculturation, Immigration, Southeast Asians, Hmong, Lao, Khmer, Cambodian, Vietnamese, Minnesota

## Abstract

**Background:**

Southeast Asian communities in the United States have suffered from high rates of tobacco use and high rates of chronic diseases associated with firsthand and secondhand smoking. Research is needed on how best to reduce and prevent tobacco use and exposure to secondhand smoke in these communities. The objective of this study was to examine how tobacco use patterns in Minnesota’s Southeast Asian communities have been shaped by culture, immigration, and adjustment to life in America in order to inform future tobacco control strategies.

**Methods:**

The study consisted of semi-structured interviews with 60 formal and informal leaders from Minnesota’s Hmong, Khmer (Cambodian), Lao, and Vietnamese communities and incorporated principles of community-based participatory research.

**Results:**

Among Khmer, Lao and Vietnamese, tobacco in the homeland was a valued part of material culture and was used to signify social status, convey respect, and support social rituals among adult men (the only group for whom smoking was acceptable). Among the Hmong, regular consumption of tobacco was unacceptable and rarely seen until the civil war in Laos when a number of Hmong soldiers became smokers. In Minnesota, social norms have begun to shift, with smoking becoming less acceptable. Although older male smokers felt social pressure to quit, smoking functioned to reduce the stress of social isolation, economic hardship, prior trauma, and the loss of power and status. Youth and younger women no longer felt as constrained by culturally-rooted social prohibitions to smoke.

**Conclusions:**

Leaders from Minnesota’s Southeast Asian communities perceived key changes in tobacco-related attitudes, beliefs, and behaviors which were embedded in the context of shifting power, status, and gender roles within their communities. This has practical implications for developing policy and interventions. Older Southeast Asians are likely to benefit from culturally-tailored programs (e.g., that value politeness and the importance of acting in ways that benefit the family, community, and clan) and programs that work with existing social structures, as well as initiatives that address smokers’ psychological distress and social isolation. Leaders remained uncertain about how to address smoking uptake among youth, pointing to a need for additional research.

## Background

Southeast Asian communities in the United States have suffered from high rates of tobacco use among men and high rates of chronic diseases associated with firsthand and secondhand smoking [1–3]. Minnesota is home to some of the largest populations of Hmong, Khmer (Cambodian), Lao and Vietnamese refugees and immigrants in the U.S. Rates of smoking in Minnesota’s Southeast Asian communities have been high among men (except Hmong men) and rates of secondhand smoke exposure have been high for all community members [4, 5]. For example, in a survey conducted in Minnesota from July 2006 to March 2007, smoking rates were 41.6% among Vietnamese men, 13.8% among Cambodian men, 31.5% among Lao men and 11.8% among Hmong men [4]. By comparison, 2010 data from the World Health Organization estimated rates of smoking tobacco use for men to be 40.0% in Vietnam, 57.4% in Cambodia, and 56.1% in the Lao People’s Democratic Republic [6]. One might speculate that these lower rates of smoking among Cambodian and Lao smokers in Minnesota, relative to rates in Cambodia and the Lao People’s Democratic Republic, could be due to more negative social norms regarding smoking and attitudes toward smokers in Minnesota. It is unclear why the same pattern was not found for Vietnamese men, although it could be the case that the population reached in the Minnesota study was different than the population studied in Vietnam [4]. To date, there is scant research on the contributors to smoking in these communities and on effective ways to reduce and prevent smoking and exposure to secondhand smoke.

We, a team of Hmong, Khmer, Lao and Vietnamese community members and academic researchers, partnered to undertake a formative community-based participatory research (CBPR) study using qualitative methods to examine the causes of tobacco use within these four Southeast Asian groups, to set the stage for future projects aimed at preventing and reducing tobacco use. We chose to use a CBPR approach because the funding agencies did not want researchers to develop intervention strategies that would be imposed on these ethnic populations, but rather to conduct formative research to identify potentially viable and culturally meaningful approaches that would be of interest to these populations. This paper reports on one important aspect of our study – examining how the experience of living in Minnesota has influenced tobacco use patterns across these four Southeast Asian groups. We began with the fundamental belief that to fully understand present day tobacco use within Hmong, Khmer, Lao and Vietnamese communities in Minnesota, it would be essential to understand how community members view indigenous (in the homeland) and recently formed (in Minnesota) culturally influenced practices of tobacco use.

There are several reasons why the experiences of these Southeast Asian community members might influence tobacco use. First, Khmer, Lao and Vietnamese populations came to Minnesota as refugees and immigrants with tobacco use already well engrained in their cultures, in which the use of tobacco was normative for men [7–9]. Khmer, Lao and Vietnamese people have been using tobacco since the seventeenth century when Portuguese and Dutch traders began distributing it as a gift to promote friendship and as an item to exchange for local goods. Tobacco use spread in Southeast Asia because it was incorporated into daily customs and rituals as social currency, and because of its purported medicinal properties. In the late nineteenth century, Europeans, especially the French colonials, began selling manufactured cigarettes in Southeast Asia and promoting them as a luxury good [10].

It is important to note that the Hmong differed in key ways from the Vietnamese, Khmer, and Lao populations of refugees and immigrants that were resettled in the U.S. The Hmong began arriving from Laos as refugees in early 1976 [11]. Their families lived as ethnic minorities, known as “hill tribes” and survived in remote areas for generations by practicing shifting cultivation of food crops and tobacco, raising livestock, and gathering food in the forests [12]. Unlike the other three groups, the Hmong started their lives in Minnesota with low levels of tobacco use. However, since living in Minnesota, more and more Hmong have taken up tobacco use, especially Hmong men and Hmong youth of both genders [4].

Second, many people in these three ethnic communities, along with many Hmong, have experienced cumulative stress which is a risk factor for tobacco use [13]. Cumulative stressors include the past trauma of prolonged periods of civil unrest, war, occupation and authoritarian rule and post-migration stress, including unemployment, language difficulties, family conflict and discrimination [14–17]. Third, many Southeast Asian youth who grew up in the U.S. have described their substance use, including tobacco, as stemming from the stress associated with the large intergenerational gap between them and their elders, [18] and the use of tobacco among their friends and family members [19, 20].

To date, most of the research examining smoking among immigrant communities has been guided by the framework of “acculturation”, which is typically defined as “the process by which foreign-born individuals adopt the values, customs, norms, attitudes, and behaviors of the mainstream culture” [21]. Studies conducted with members of Asian American and Latino immigrant communities have found a higher likelihood of smoking among highly acculturated women and youth, and a lower likelihood of smoking among highly acculturated men [21, 22]. These findings also are consistent with global trends in smoking behavior in which women’s rates of smoking tend to increase as societies become modernized and as gender equality increases [23].

Recently, the use of acculturation as an explanatory framework for understanding health behaviors, and the ways in which acculturation is typically operationalized have come under increasing scrutiny. Critics have argued that the acculturation framework ignores important historical, economic, and political contexts (e.g., circumstances of migration); ignores ethnic heterogeneity; and simplifies the process of adjustment as one of swapping American or “Western” norms for “traditional” norms. For example, many studies using acculturation as an explanatory framework treat culture as an individual-level variable rather than one at the level of the family or community. Some have argued a family- or community-perspective is particularly important when studying collectivist or group-oriented societies (e.g., Southeast Asian societies) [24–27]. Moreover, while the extant research provides important information about the patterns of tobacco use among different ethnic minority subgroups, it provides fewer insights into the meanings and significance of tobacco within these communities.

To lay the groundwork for developing sound tobacco control policy, researchers and policy-makers have called for studies that examine historical and cultural factors that influence these ethnic groups’ present-day tobacco use norms and practices, with an emphasis on examining community-level factors rather than individual-level factors [28–30]. The present study aimed to produce an in-depth understanding of tobacco use within Minnesota’s Hmong, Khmer, Lao and Vietnamese populations within the context of the experience of resettlement, immigration and adjustment in order to inform the development of interventions that would reduce the harms of tobacco in these communities. To this end, we conducted this study using qualitative methods to explore the role of tobacco in Hmong, Khmer, Lao and Vietnamese cultures in Minnesota.

## Methods

This study was approved by the Institutional Review Board of the Minnesota Department of Health.

We used principles of community-based participatory research (CBPR) in which community members, representatives from different community-based organizations, and researchers collaborated throughout the research process. In 2002, ClearWay Minnesota^SM^ and the Center for Prevention at Blue Cross and Blue Shield of Minnesota began engaging in dialogue with representatives from the Asian Pacific Tobacco-Free Coalition of Minnesota (APT-FCM), the Southeast Asian Refugee Community Home (SEARCH) and several other local advocacy and social service organizations that serve Southeast Asian communities in Minnesota, along with several university-based researchers. These groups formed the multidisciplinary Diverse Racial Ethnic Groups and Nations (DREGAN) Southeast Asian Community Advisory Committee. Representatives from these groups were involved in establishing the research project and all subsequent phases of the research including: defining the research questions and developing the interview guide; identifying, recruiting, and interviewing community leaders; analyzing data; validating and presenting results; and dissemination of the findings. To undertake the study, the Southeast Asian Community Advisory Committee designated a research team (the DREGAN team) made up of Hmong, Khmer, Lao and Vietnamese community members and academic researchers (not of Southeast Asian origin).

We adhered to most of the principles of CBPR with two exceptions: the decisions to focus on tobacco and the basic study design (i.e., interviews with community leaders) were driven by the two sponsoring organizations, ClearWay Minnesota and the Center for Prevention at Blue Cross and Blue Shield of Minnesota. The decision to focus on tobacco was based on the funding agencies’ shared missions to work on tobacco control. The decision to interview formal and informal community leaders was arrived at pragmatically in consultation with the community advisory board because community leaders were seen as experts on the social norms related to tobacco use in their communities and because they were in a position to play a key role in changing these norms and to elevate the importance of tobacco use as a community problem.

The DREGAN team identified domains of inquiry that would be meaningful for Southeast Asian community members to use in future tobacco control work. These domains included leaders’ own perceptions of tobacco, leaders’ perceptions of community members, leaders’ perceptions of tobacco use in their communities, and leaders’ perspectives about prevention. The DREGAN team then developed four overarching questions that guided the study: 1) What were the community leaders’ own feelings, views and beliefs about tobacco use and secondhand smoke? 2) What did the community leaders think members of their ethnic group in Minnesota believed and perceived about tobacco use and secondhand smoke? 3) What patterns of tobacco use and secondhand smoke exposure did the community leaders observe? 4) What did the community leaders think might be done to reduce and prevent tobacco use?

To answer these questions, the DREGAN team developed an interview guide and translated it into Hmong, Khmer, Lao and Vietnamese. Team members with research expertise conducted trainings on semi-structured interviewing techniques for the Southeast Asian research team members who were fluent in English and their native language. The guide included broad questions about tobacco use, rather than just cigarette smoking, including a question that explicitly asked about “other forms of smoking including cigars, chewing tobacco and/or betel nuts, and pipes”. The Community Advisory Committee provided input and feedback throughout the process of developing the interview guide.

In this study, we used a nonprobabilistic sampling procedure to recruit participants with the intention of gathering in-depth information about shifts in cultural patterns rather than generalizing to a larger population in terms of rates or proportions. We used a stratified purposeful sampling frame [31] to select specific subsamples for comparison. We defined the total population of interest as individuals who identified themselves as being “Southeast Asian” and residents of Minnesota, including refugees, immigrants and adults born outside of Southeast Asia with at least one Southeast Asian parent. Within this population, we included four Southeast Asian groups according to ethnicity: Hmong, Khmer, Lao and Vietnamese. Within each of those four ethnic subpopulations, we selected a class of individuals our community advisory committee considered to be informal and formal leaders who were highly knowledgeable and articulate. We stratified the sample further by gender to include 8 males and 7 females (60 leaders in all).

With each participant, we conducted a semi-structured one-to-one interview in whatever language or combination of languages the participant felt comfortable using. Each one-to-one interview lasted from 60–90 minutes and focused on tobacco-related behaviors, knowledge, and norms in the respondent’s land of heritage and in Minnesota. Before the interviews were conducted, written informed consent was obtained from each participant. Interviews were audiotaped and then transcribed. After the interview, each participant completed a brief demographic questionnaire and received a nominal gift for his or her participation.

Our team analyzed the data collected through the interviews. One bilingual team member, usually the interviewer, typed the audiotaped interview verbatim, in the language in which it was conducted and, when needed, translated it into English. A second bilingual team member independently compared the English transcription against the transcription in the original language, looking for errors. Any differences in translation were discussed and resolved between them. For the interviews conducted in English, we kept the original grammar of the participants, even if it was grammatically incorrect. We then undertook our analysis in three phases. In the first phase, we used a parallel method of analysis in which cultural insiders in our team analyzed the original transcripts, most of which were in their native language while cultural outsiders on our team analyzed transcripts in English. Through these parallel readings, we conducted a standard ethnographic analysis of coding transcripts and sorting text segments into topic documents. We classified text iteratively according to an evolving coding scheme as topics emerged. Examples of codes included gender, recollections about tobacco use in one’s land of heritage, smoking status, and secondhand smoke exposure. We achieved a consensus among team members in coding by resolving discrepancies through discussion about different interpretations.

Team members then analyzed text segments in each topic document to identify major themes, and to note the degree of variation in views related to these themes. We were sensitive to the amount of variation among participants on a given topic (between and within ethnic groups), and we identified variation as part of our coding, noting disconfirming cases. Specifically, as part of our analysis protocol we examined minority/contrasting views and examined whether there were differences by number of years in the country, smoking status, community, and gender.

In the second phase of our analysis, we used a standardized framework for ethnographic analysis called a face sheet comparison to look at each interview as a whole and to compare interviews to find similarities and differences between the participants. We identified topics for which there was substantial thematic similarity among all 60 participants. Then, we identified 11 topics where the data revealed a wide range of viewpoints, opinions and levels of knowledge. Topics included attitudes about smokers, ideas about addiction, and beliefs about the effects of smoking on others.

We sought to interpret the themes we identified within the cultural and social context of Minnesota, fusing Southeast Asian team members’ insider knowledge with other team members’ research expertise. Some of our analysis focused on identifying differences between subgroups, including by gender and language proficiency, using this as a proxy for level of acculturation to American norms. Throughout our analysis, we retained the diversity of experiences, opinions and ideas in the data to avoid oversimplifying the complex reality that participants presented in the interviews, and to avoid stereotyping.

In the third phase of our analysis, we conducted four separate meetings with members of each of the four communities (which included some interviewers and participants) in which we presented and then extensively discussed the major themes and variations we heard and our research team’s interpretation of these results. This “member-checking” process resulted in clarification and revision of our initial findings, and ensured that our conclusions accurately reflected the experiences and perspectives of the participants.

## Results

Participants included students, retirees, journalists, program directors, religious leaders, counselors, healthcare workers, teachers, social workers, volunteers, community advocates and heads of community organizations. Table 1 presents participants’ age, smoking status, and years in the United States broken down by gender and ethnicity.

**Table 1:** **Participant characteristics by ethnicity and gender**

	Hmong		Cambodian		Vietnamese		Lao	
	Men (n = 8)	Women (n = 7)	Men (n = 8)	Women (n = 7)	Men (n = 8)	Women (n = 7)	Men (n = 8)	Women (n = 7)
	M(SD)		M(SD)		M(SD)		M(SD)	
Age	43.9 (15.1)	32.7 (7.9)	56.7 (8.8)	46.3 (5.9)	60.3 (2.1)	34.0 (14.0)	55.7 (13.1)	39.2 (14.8)
Years in U.S.	17.0 (4.3)	21.4 (3.7)	19.2 (8.0)	19.0 (6.2)		—*	17.7 (4.3)	19.8 (5.9)
Smoking status (%)								
Current	12.5%	0	0	0	16.7%	0	12.5%	0
Former	25.0%	0	75%	0	33.3%	0	75.0%	0
Never	62.5%	100%	25%	100%	50.0%	100%	12.5%	100%

*Missing data.

Generally, we found similarities among the Khmer, Lao and Vietnamese participants’ accounts of how tobacco was used and regarded in their lands of heritage. The Hmong participants’ accounts were quite different from the other three groups, indicating that Hmong in Southeast Asia used and regarded tobacco differently from the other three groups. Accordingly, we present the findings from the Khmer, Lao and Vietnamese participants separately from the Hmong findings. Additionally, in presenting our results, we have chosen quotes that are highly representative of the participants’ views about a given theme.

### Tobacco-related beliefs, customs, and norms

#### Tobacco as a valued part of material culture

According to nearly all of the participants, manufactured cigarettes were a highly valued part of material culture in their lands of heritage. The participants described how manufactured cigarettes were an integral part of gift-giving at weddings, funerals, and other ceremonies, and how men used them in social encounters to forge and strengthen bonds. A Khmer male participant explained that tobacco (especially cigarettes) was “about guests greeting, making a connection, building relationships, offering to each other…”

A Vietnamese male participant recalled a saying that “…a conversation had to begin with offering tobacco”. In homes, a host who failed to offer a male guest a cigarette would be indicating that she/he “…does not really want to welcome the visitors” (Lao male). Khmer, Lao and Vietnamese participants recalled that people used tobacco to foster warm feelings of camaraderie, as one male Lao participant explained,

…When there was a festival or a time when you went to the temple or in someone’s home after the meal, in our culture older people would gather and start to smoke tobacco… Getting together [and] then smoking …[was] the happiest time they would have, and it symbolized the unity of the people…

In Southeast Asia, tobacco brands and forms of smoking tobacco became symbols of social class. Affluent Khmer, Lao and Vietnamese smokers and those aspiring to be affluent chose to smoke expensive European and American brands to indicate their high social status. A male Vietnamese participant explained,

Smoking shows off a person’s character, wealth, status, makes people look “cool”. People’s socio-economic class is reflected in what they smoke. Poor people/peasants smoke waterpipes, the majority smoked hand-rolled cigarettes, and rich people smoked imported cigarettes or cigars.

Similarly, a male Khmer participant recalled,

The American name brands or any imported cigarette such as Winston, Salem… They made the smoker look like a high-class person. The elite who held employment positions in offices and smoked those imported cigarettes inspired me. It symbolized a status of being high-class. It inspired me.

#### Tobacco use, age, and gender

Tobacco was used in Khmer, Lao and Vietnamese societies to create and sustain social arrangements among adult males and to reinforce gender ideology. The act of smoking also was bound up with sensibilities about masculinity and seen as part of manhood. According to an elderly Khmer man, “…when I was a single youth, if you didn’t know how to smoke or drink you were not a man…” Likewise, many participants, such as one Vietnamese man, described smoking in terms of a rite of passage that distinguished boys from men. “… Besides, when a boy wanted to show that he was mature, he smoked … to show that he was a grown-up, that he no longer was a boy. And … sometimes, [boys] wanted to show they fully lived their own life”.

The participants described how families and communities forbade children younger than 18 and women from smoking. Elderly men and women chewed tobacco with betel nuts. A Vietnamese man explained the disdain people had for any young person seen smoking, recalling “…if people see a youth smoking, they would perceive it as something bad, as a thorn poking their eyes”.

A Lao woman explained,

Smoking in my native land is considered a normal thing to do for adults or the man of the house. For young adults, they would be considered bad people. In order for it to be acceptable, young adults have to be at least 20 years or older. Otherwise, parents have the right to punish them.

Discussing the strong prohibition against women smoking, a Khmer man exclaimed:

If you were a female and smoked, that woman was not good. That was a city woman. And that was a woman who had no good occupation, you see. The woman who smoked …[was] a woman they don’t want to associate with…. The woman was…in a bad part of society…

#### War and smoking

Participants recalled an increase in smoking during the period of the civil wars in Cambodia, Laos and Vietnam and in the period of socioeconomic instability thereafter. One Vietnamese participant became a smoker after he joined the army due to “peer pressure” and as a way to cope with fear and to “relieve tension”. Another Vietnamese man believed that social unrest and unemployment after the fall of Saigon in 1975 led to an increase in smoking, explaining, “Before 1975 people smoked because people wanted to imitate each other and show-off… But after 1975, the number of smokers even increased for the following reasons. The unemployment rate was high. When people are unemployed, they smoke more”.

Several Khmer participants described how the Khmer Rouge regime’s harsh rule and tactics caused an increase in smoking. According to some, the Khmer Rouge distributed cigarettes in labor camps to keep people alert, to enhance productivity, and to show that everyone was equal. One participant remarked, “I saw a dangerous act committed by the Khmer Rouge. They distributed cigarettes to the children… Children and women smoked openly”. Another Khmer participant explained that under conditions of forced labor non-smokers took up smoking because it was the only time that they would be able to take a break from work. Others described how people became smokers so they would be given valuable cigarettes that could be traded for food, or to keep away the malaria-carrying mosquitoes. Under the Khmer Rouge, many also turned to cigarettes for solace:

… During Khmer Rouge power in our country we had a hard time. Lots of people learned to smoke, including myself…We believed smoking will keep the mosquitoes away at night.…Sometimes, we taught our children to smoke by ourselves… leading them to a smoking habit… And sometimes, some other people thought that smoking can make them relax.

#### Tobacco, medicine and health

Khmer, Lao and Vietnamese in Southeast Asia valued tobacco for its purported medicinal properties. Some participants associated tobacco use with health and cure for ailments. For example, a Khmer female participant described a myriad of medicinal uses for tobacco. “…[Tobacco] was used to remove leeches, cure skin diseases or stop the itch caused by insect bite, to kill germs, cure abscess, stop bleeding, clean teeth, use as a band aid, heal a sprained ankle and to heal a snake wound (or slow the effect of the venom)”. A female Vietnamese participant was careful to distinguish medicinal tobacco from the act of smoking, stating, “The tobacco used as medicine to cure diseases, when people smoke or … put on wounds to stop bleeding…We can also call this way of tobacco use as medicine, but… when you smoke tobacco, inhale it into your lungs and call it medicine – that’s never true”. Participants recalled that in Southeast Asia most people had little knowledge of the risks that smoking posed to health. Particularly in rural regions, very few people had any understanding of Western biomedical knowledge about tobacco use.

#### Tobacco use in the land of heritage among the Hmong

In contrast to the Khmer, Lao and Vietnamese participants, the Hmong participants described how regular consumption of tobacco was unacceptable, disparaged, and rarely seen in Hmong villages. Unlike the other groups who lived under French colonial rule and were exposed to French norms and patterns of smoking, the Hmong lived in remote highland villages and were relatively isolated from French colonial influence. Smoking was only seen among some elders who smoked tobacco hoping to relieve arthritis and pain. One female Hmong participant recalled that Hmong smoked tobacco from a waterpipe to treat “…body ache or arthritis or waist pain or stomach pain…”. Like the other participants, a Hmong man recalled that Hmong people had very little knowledge about the health effects of tobacco saying, “In the old days of our time, people never heard of cancer or heart disease”.

Several Hmong participants’ depictions of smokers were extremely negative. They described smokers as lazy, immature, and hurting their family’s reputation: “[the] majority of the people thought that the ones who smoked … [were] not considered good human beings, or did not seem mature; that is why the person got into smoking. Elders viewed that as part of the culture and put smokers into the position that they were not worthy”. Other Hmong participants observed that although smoking tobacco was not encouraged, the real vitriol was reserved for opium smokers. Despite differing views as to the *extent* to which tobacco smokers (as opposed to opium smokers) were stigmatized, several Hmong participants discussed various ways in which villages would sanction or ostracize smokers.

… If your son or daughter wanted to marry someone who was smoker in the village, the whole villagers would recommend that you not marry that person. The villagers tend to exclude that individual from the society. Whether the person is male or female it will be difficult to look for a mate…if I have sons or daughters who are smokers, or if I have a son who wants to marry a female smoker, I personally would not recommend him marrying her because she does not have good character. I don’t know if some smokers are nice too, but most smokers are not good people. Everyone perceives that way about people who smoke and would refuse to accept that person.

Hmong people’s existence changed dramatically during the civil war in Laos when, in exchange for protection by the United States from their enemies, many Hmong fought the Communist insurgents on behalf of the U.S. During that time, a number of Hmong soldiers became smokers when the Central Intelligence Agency (CIA) gave them American cigarettes to help them stay awake. Some Hmong elders blamed smoking on the “American War” and believed that sons and nephews of men in the military smoked to emulate their fathers, “spreading” the norm of smoking throughout the Hmong community. For example, when we asked a Hmong female participant about her image of a Hmong smoker she replied,

You know the first image that comes to my mind is… you think about what kind of family they grew up in and how they start smoking or were they people that always in trouble or people that just pick up smoking as a habit from friends and family or the war.

#### Tobacco use in Minnesota

The participants described many ways in which tobacco use patterns, beliefs, norms and practices changed among their ethnic groups living in Minnesota. Several participants observed that some in their ethnic group had started smoking after arriving in the U.S. These participants felt that some people had taken up smoking because of decades of accumulated trauma, dislocation and stress, a view that is supported by evidence that many Southeast Asian refugees suffer from post-traumatic stress disorder and depression [32]. As one Khmer male explained,

Back in the homeland, there was a war, the sufferings, and hardship… When we are here, we have a lot of stresses. Some people came here without knowing any English. They don’t speak English. They don’t know how to drive and cannot go out like others so they are always staying home like the rabbit in its cave. When they stayed inside for so long, they will have a lot of stresses and they don’t have any other ways…but pulling cigarettes to smoke…

Many participants explained that members of their ethnic group had found that, unlike in Southeast Asia, in Minnesotan society in general smokers do not derive high social status from their smoking and are somewhat ostracized, which is itself another stressor. One male Khmer participant explained the contrast in smokers’ experiences saying,

…The difference is [that] in Cambodia you could smoke if you wanted to. That was normal, you see. Nobody was criticizing you… But here, we are not welcoming those who smoke… When they need to smoke, they smoke alone, you see.

Khmer, Lao and Vietnamese refugees and immigrants also confronted a dramatic difference in the way in which smokers were portrayed. Unlike in Southeast Asia, where smoking imported and manufactured cigarettes signified high social status, smokers’ experiences in Minnesota were dramatically different. As one Khmer male explained,

…in Minnesota, most people, young and old, have perceived the harmful impact of smoking. So, if anyone keeps smoking, he or she should be classified as unlikable person and ignorant. Society would not accept this person. In Cambodia, a smoker’s value depends on the value of cigarettes. It seems that the expensive cigarettes ennoble the smokers into a high-class status…In this country, no matter how high the price of cigarette is, the value of smokers may not be as high as their cigarettes.

Despite the perception that people in the general Minnesotan population tend to look down on smokers, the majority of the Khmer, Lao and Vietnamese participants believed that only smokers who smoke around others are regarded negatively. Several female participants described how their perceptions of smokers had changed since they came to Minnesota. They expressed their strong personal aversion to the unpleasant smell and appearance of smokers, and they associated smokers with sickness. For example, a Vietnamese female participant described smokers as having “a dirty and unhealthy image. Smokers and everything surrounding them are so smelly…”. Several other participants perceived smokers negatively because they felt that smokers were harming innocent family members.

Most participants talked about how Southeast Asians in Minnesota had established new social norms governing smoking. For the participants, one of the most striking differences between patterns of tobacco use among Southeast Asians in Minnesota versus what they recalled in Southeast Asia was that in Minnesota, smoking was becoming common among youth and women, two groups that were forbidden to smoke in Southeast Asia. Although Khmer, Lao and Vietnamese participants believed that smokers were generally viewed more negatively in the U.S. than in Southeast Asia, those same participants, as well as some Hmong participants, viewed the U.S. as a place where all people had the freedom to smoke, regardless of age or gender. They constructed “freedom” in this context, as individual autonomy to do what one wishes. This construction of personal autonomy was particularly appealing to women and young people. As a Vietnamese woman lamented,

…when our Vietnamese people immigrated to the United States, we had communication and exchange with the American world…we have learned many good things, but we have also learned a lot of bad things … the young ladies nowadays seem to adapt the concept of freedom, so they would think they should have the right to smoke just as their brothers, and would not worry about their reputation…

Some participants saw the greater “freedom” to smoke as a function of immigrating from close-knit communities where if you smoke “you are worried about other people telling on you” (Hmong woman) to large urban areas with greater anonymity. As a Khmer participant bemoaned, “[Smoking among youth]…is not acceptable in our community but the parents couldn’t do much because of the convenient access to cigarettes. They [youth] can buy it or get it from friends. This is becoming a part of the parents’ stress”. Similarly, a Hmong woman said, “I can start to see women smoke tobacco because, I think, in Laos, if you see a woman smoke, people would talk about it, and then that person would feel ashamed of smoking, but in this country and in Minnesota, like the mainstream culture, women, children, everybody smokes…” A Hmong woman captured the effect of anonymity on smoking in a vignette she described,

The other day I was at a park and we ran into a family and the husband was smoking and when we walked around the park, we ran into the family again and the wife was smoking too… She was in her late thirties and I thought wow! That’s a surprise because she’s speaking Hmong to her kids and that’s really rare. Now if she was in Laos because it was more restricted and more looked down upon by the elders…she would not have been smoking.

A few participants characterized their ethnic communities as being in a “state of flux” in which some members want to hold onto the Southeast Asian values they grew up with whereas others want to adopt American ways. It appears that community members who adapted more readily to mainstream American society and who realized that others in their community disapproved of their smoking negotiated this potential hazard by refraining from smoking in their homes and around community members, and instead restricted their smoking to their peer group. For example, a Hmong woman explained,

I don’t think it’s acceptable to…have elders and parents see youth smoking. I mean, that is still looked down upon and it’s still a bad reputation…because…they won’t want other member of the Hmong community looking down on them … But I do have …some siblings, younger siblings that would smoke but they won’t smoke at my house just because they respect me and won’t want to make me lose face…

Most participants reported that although some smokers in their ethnic group still smoked at social gatherings, generally people in their ethnic group had become less tolerant of smokers smoking around others, particularly around children, pregnant women, elders with health problems, and in the homes of non-smokers. As a Vietnamese former smoker explained,

…smoking at social gatherings still occurs but smokers are expected to smoke away from nonsmokers. Yes, tobacco is still used in our celebrations. But, at many places, smokers are asked to sit separately. Many non-smokers do not like to smell the cigarette smoke.

Several participants stressed the importance of respecting the new norms in America explaining how sensitivity and respect for others were important cultural values among Southeast Asians. As one Khmer man explained,

As our ancestors taught us, we should follow the winding stream when we enter the river’s journey. In America, there are some restrictions for smoking. The dangerous impact of smoking is often publicized in TV. We understand it… Therefore, in the wedding ceremony, where we used to serve guests with cigarettes, we offer candies as a symbol of thanksgiving. I had done it in my daughter’s wedding. I encountered some objections, but I went ahead using candies instead of cigarettes. It’s less harmful.

Some participants attributed the differences in norms to the effect of different climates in Southeast Asia and Minnesota, as well as to the different housing conditions. Whereas people in Southeast Asia lived in a tropical climate in houses designed to be open and naturally ventilated, in Minnesota people live in houses designed for hot and cold climates with air conditioning and heating, with minimal natural ventilation. In such conditions in Minnesota, Southeast Asians found that smoking indoors was inconsiderate. However, several participants explained that it is also considered rude to tell a guest that they cannot smoke in one’s own home. As such, guests must intuit the appropriate rules because they are not stated outright. As one Hmong participant explained,

… the person who smokes has to know …that in order to respect the family that they go visit not to smoke in their house, because the host family or the family who … invite you to go to their house, they’re not going to tell you that… Even though it’s not acceptable in their house they’re not going to say directly to that person, that would be … disrespect toward their visitor, so the visitor has to know … by him or herself that it’s not acceptable…

#### Limited increases in awareness of health risks

Participants described the contrast between living in Southeast Asia where, at the time, people had little knowledge about the effects of tobacco on health, versus living in Minnesota where people have been exposed to messages about the health risks of firsthand and secondhand smoke for decades. One Vietnamese male participant noted that in Minnesota, “…the television always broadcasts noisily that smoking causes this, causes that…” Some of this information had reached Southeast Asians living in Minnesota. When we asked a Hmong participant about the effects of smoke on nonsmokers, he said, “I know that they have commercials on TV that it’s bad. You can still develop lung cancer or other health problems just by secondhand smoke”. However, many participants emphasized that despite the distribution of health information through various media in Minnesota, many Southeast Asians still lacked awareness about the dangers of smoking, especially those who did not read or understand spoken English, such as elders. As a Hmong leader explained, “everyone seems to hear rumors that smoking is not good, but they do not know exactly what it is that is not good”. Similarly, a Lao participant explained,

…most of our people don’t know what caused them to be sick. When Mr. X or Uncle Y got sick they knew that he had lung cancer … [but they had] no explanation to what caused that… Nobody would say that it was because he had been smoking for over twenty years. Even his family wouldn’t say he had been smoking for over twenty years [and] that’s why he has cancer.

Knowledge about the health risks of secondhand smoke appeared to be limited in all four ethnic groups. For instance, when we asked a female Hmong participant about the effects of smoke from another person’s cigarette, she answered, “…The one that doesn’t smoke will probably not have any problems in their body and health because that person is not smoking”. However, somehow, nearly all of the participants seemed to have learned that secondhand smoke exposure is harmful for children, pregnant women, and the elderly. As one Vietnamese participant explained,

…even with the current publication of the numerous flyers on smoking and secondhand smoking, this issue is not a real concern for the Asian people in general. Except this specific situation, if the smoker has a family with young children, it would be better for the children’s health if the smoker does not smoke in the house.”

Additionally, some participants were misinformed about the effects of smoking tobacco. Several held the belief that homegrown tobacco (produced “without chemicals”) and tobacco smoked through a waterpipe were safer than manufactured cigarettes. Some participants indicated that community members pointed out that some elders had smoked all of their lives without getting sick. For example, a Lao participant explained how people’s perceptions about the effects of smoking were heavily influenced by the events they observed, stating,

Some people quit for five to six months and then died of cancer or have diabetes …I had seen three or four friends who died because they quit smoking rapidly. Within five to seven months of their quitting, some people have diabetes then cancer without any reason, so it’s hard for people to decide to quit.”

#### Increased motivation to quit among older men

Immigrating to the U.S. along with increased pressure from family members sparked some Southeast Asians’ desires to quit. A Vietnamese woman, for instance, estimated that almost fifty percent of Vietnamese smokers in the U.S. want to quit because “…they know that their health is very important here. If they cannot go work, how could they pay the bills?” A Khmer man recalled that the shame and disgrace he experienced as a smoker in the U.S. led him to quit:

…I used to smoke before because I used to work as a constructor to build roads, bridges… [my company] had its own good rules [in] which they don't allow the smokers to smoke in a working place. They allowed them to smoke outside. Then I felt ashamed. …And I understood that when they [my employers] do good things, I have to respect them and follow them. And it is not only the benefit to the community but to [the] individual. That is why I decided not to smoke anymore.

Several participants recounted incidents of smokers quitting in response to direct or indirect criticism from their family, community, and larger society. They recalled that wives and children pressured male smokers to quit – events that would be much less likely in Southeast Asia where patriarchal family hierarchy was more rigid and respected. According to a Vietnamese woman, “whenever I see my children or my husband touch a cigarette, I start complaining. I complain all the time. I say, ‘Why do you want to smoke? It’s costly, and it’s harmful to our health.’ So all of my family members had to stop smoking”.

Despite such pressure, some smokers simply changed their smoking patterns in response to social disapproval, rather than quitting. A male Khmer participant described it this way,

…When a husband smokes, the bad odor disturbs his wife. She often discourages him from smoking. And her attitude of dislike grows. It is a disgrace for a husband. As for the youths, they would have encountered the same situation: disgraced by the elders. Therefore, both youths and general individuals are afraid of losing face and respect. Then they would not smoke in public. They would do it in a private place so that other people in the community cannot see them, and they cannot look down at them.

While male Khmer, Lao and Vietnamese smokers faced increasing pressure to quit, the Hmong participants explained that Hmong were reluctant to criticize male smokers. For this reason, Hmong participants felt that their ethnic group was more concerned about dealing with the increasing numbers of young Hmong who were taking up smoking.

## Discussion and conclusions

This formative study adds to the literature by documenting the effects of immigration and acculturation on tobacco use among Southeast Asians in the U.S. by exploring cultural and contextual factors that contribute to changing patterns of tobacco use [21]. These findings suggest potential directions for public health policy and practice.

Our results help explain a consistent finding of prior studies documenting higher rates of smoking among acculturated women and youth, and a lower likelihood of smoking among acculturated men. Our research identifies several contributors to these shifting patterns of tobacco use. First, most adult community members – including many smokers – no longer view the act of smoking cigarettes and the identity of being a smoker as having potent, positive symbolic significance, transactional utility, and social acceptability that they once did in Southeast Asia. Now that these Southeast Asians are living in Minnesota, while there has not been a complete reversal of this view, there has been a gradual shift such that cigarettes have less symbolic and practical significance. The information from the participants suggests that a large majority of Khmer, Lao and Vietnamese older community members in Minnesota have changed their perceptions about tobacco. This has contributed to an emerging set of social norms that govern tobacco use among many, although not among some youth and some women. Smokers in these communities have contributed, in varying degrees, to producing these cultural shifts by changing their own smoking patterns. We found that the pattern of change among Hmong community members was different from that of the other three groups because Hmong came to the U.S. with less favorable perceptions about tobacco and less approving norms of tobacco use. Still, Hmong too face rising rates of youth smoking.

In adjusting to life in a new context, the primary message many refugees and immigrants have received is that smoking is generally unhealthy and not widely accepted socially. Community members have encountered images and information in English language media and in their own ethnic media that are vastly different from what they were exposed to in their lands of heritage, and they now live in a context that has much more restrictive laws and regulations regarding tobacco use. Moreover, social norms in Minnesota restrict smokers considerably compared to social norms in Cambodia, Laos and Vietnam.

The Khmer, Lao and Vietnamese participants felt that sizable majorities of their communities no longer see smoking as an essential part of ceremonies and greeting rituals. Whereas it was once considered essential to offer guests cigarettes as gifts at social occasions to create a pleasant, relaxing ambiance and enliven the “happiest times” to bring people closer together, in Minnesota, community members mainly offer other party favors to guests such as candies. In doing so, they demonstrate their sensitivity for nonsmokers and awareness of smoking as an unfavorable, not-family-friendly social activity. Even within the Khmer, Lao and Vietnamese men’s worlds, the pattern of exchanging cigarettes as a token for cultivating close social relations is in decline. When men were in Southeast Asia and meeting each other in business situations and in male-only interactions (e.g., military), they handed each other cigarettes to forge a “connection”. In Minnesota, men have been abandoning this social ritual because fewer men smoke, because smoking no longer has the same association with masculinity, and because expensive brands no longer has much cachet.

Our results suggest that in Minnesota in the context of private homes, now many Khmer, Lao and Vietnamese community members no longer offer cigarettes to guests or potential associates. Many nonsmokers no longer find it acceptable to allow smokers to smoke in their homes. Accordingly, many smokers no longer feel that they can smoke wherever and whenever they wish to smoke in others’ homes, in other locations, and even within their own homes. Now, many smokers regulate their smoking according to the social and physical context in the moment when they have the urge to smoke. Many smokers also either voluntarily smoke outside or away from nonsmokers, or at least they ask for permission before lighting up a cigarette with the awareness that that permission may, politely, not be granted.

In addition, gender ideology about smoking has shifted. Whereas young and older men once viewed smoking as a symbol of joining men’s world and community members once expected that the majority of boys would become smokers when they entered adulthood, now in Minnesota, many family and community members – including some male smokers – discourage boys and young men from taking up smoking.

The shifts in perceptions and norms have not all been positive from a public health perspective. In all four ethnic populations, it appears that increasing numbers of adults no longer forbid girls from smoking or discourage young women from smoking. In Southeast Asia, girls and young women who smoked were often ostracized and targeted with gossip and innuendo about being a prostitute. Now, in Minnesota, fewer community members subject girls and young women to such harsh social sanctions and criticism. Although many community members still find it unsavory and objectionable to see girls and young women smoking, most tend to turn a “blind eye,” assuming that these female community members are just becoming “Americans” or becoming “Americanized” –processes that at least some community members see as inevitable as they relinquish their efforts to restrict women’s freedom [23].

For more conservative community members, the sight of youth of both genders and the sight of young women taking up smoking and smoking openly in public represents a loss of valued cultural traditions. They see these phenomena as further evidence of the undermining of social hierarchy within their ethnic groups. These phenomena also represent what they perceive to be the distortion of American values of freedom and equality that produce “out of control” behaviors and gives undue influence of their children’s friends [33]. Undoubtedly, these cultural shifts have created an opening for the tobacco companies, some of which have directed marketing executives to “…investigate the possibility of utilizing men and women and targeting youth in advertising strategies” and have disseminated research “indicating that Asian-American women are smoking more as they believe that they should enjoy the same freedom as men” [34].

For some smokers, these changes in perceptions and norms can be fraught with powerful negative emotions that are linked to larger issues related to the stress of immigration and adjustment to life in America. Although some participants (particularly nonsmokers) viewed American anti-smoking sentiments as positive, they acknowledged that some smokers and their families experience strife. They reported that their wives, children and other family members now openly and sometimes harshly pressure husbands, fathers, and other male family members to quit smoking. This confrontation had an impact not only on male smokers who feel that such pressure is an affront to their patriarchal authority within their households, but also on male smokers’ social standing within communities. Such pressure, coupled with the shifts in community-wide perceptions of social norms described above, can induce feelings of shame as smokers face implicit and explicit criticism from their family members, their community, and the wider society in Minnesota.

The participants were clear that many male smokers used tobacco to alleviate stress, anxiety, and depression, as seen in other studies, [1, 35] as they struggled with feelings of social isolation, dislocation from their own culture, lack of English language proficiency, lack of employment, and poverty. They also felt many smokers were ashamed that they could not quit, particularly in the face of pressure from their family members and negative images of smokers that had become pervasive in Minnesota. The participants were also clear that their communities and health professionals need to take care with these smokers.

There are several limitations to this research. Our findings are derived from in-depth interviews with participants who are informal and formal community leaders and thus, our findings are based on their experiences, perceptions and opinions. These leaders tended to be somewhat older on average than the general population in their ethnic communities, and by definition they had higher social status than most other community members. Additionally, many of these leaders were not current tobacco users. Thus, while they had a wide perspective based on their many contacts and activities within their communities, their viewpoints do not directly reflect those of smokers and other important segments of these communities. This may have led to comments that suggested a larger shift away from the normative use of tobacco than was the case among smokers and in the community as a whole. However, to the extent that these leaders have a disproportionately large influence on their communities, their perspectives are critical to understanding and shaping tobacco use patterns in these communities.

Over time, memory about life in one’s land of origin can fade or become distorted with nostalgic sentiments. So, particularly for those participants who had been in the U.S. for several decades and had not been back to their land of heritage since, their recollections may have become somewhat less reliable. We only interviewed Hmong, Khmers, Laos and Vietnamese in Minnesota. Thus, our findings may not be generalizable to other Southeast Asian populations. It is possible that the interviewing process induced some acquiescence bias because ethnic community partners conducted the interviews rather than professional researchers. Although the community partners received training for conducting interviews and they asked pilot-tested questions as much as possible, their interviewing technique may have inadvertently led some interviewees to bias their answers.

We also did not directly explore some other potential causes of increased smoking uptake among Southeast Asian immigrants in the U.S., such as the impact of tobacco marketing activities, particularly those directed toward women and youth [34]. Additionally, we did not explore the influence of some important contextual factors such as tobacco control policies and the demographic composition of the residential population.

An understanding of the historical events that bring refugees and immigrants to the U.S. and their experiences adapting to life in the U.S. provides a necessary foundation for understanding their cultural shifts in patterns of tobacco use. Our research has shown that Southeast Asians of different generations and life stages face different problems. Southeast Asians who are older tend to face barriers to quitting that include low levels of knowledge about health risks, limited familiarity with the concept of preventive health, social isolation, positive associations regarding tobacco, and stronger adherence to rituals involving smoking. However, older people possess many attributes that can serve as the basis for culturally-rooted tobacco control programs, such as having tightly knit families, communities, and clans, and having a high level of cultural responsiveness to social norms, including American norms. This group is likely to benefit from programs based on their culturally-rooted values (e.g., the values of politeness; the importance of acting in ways that benefit the family, community, and clan) and programs that work with existing social structures, as well as initiatives that address smokers’ psychological distress and social isolation.

It is less clear (to the participants and to us) how to address smoking uptake among the younger generations of Hmong, Khmer, Lao and Vietnamese. Studies in the U.S. have shown that Southeast Asian youth suffer from stress, anxiety and depression associated with poverty, racism, discrimination, and teasing by their peers. Also, they may feel disadvantaged by the absence of a parent who is able to guide them and advocate for them within school, and they may feel hampered by being part of an ethnic community that is somewhat set apart from “mainstream” American society [36, 37]. These stressors may be compounded by the intergenerational conflict that ensues as youth – particularly girls and young women – adopt “American” behaviors that are prohibited by their ethnic group’s traditions. Young people tend to perceive smoking as a stress release, and stressors such as discrimination are associated with smoking behavior [38]. It is critical that tobacco control programs be developed that address these types of stressors that are likely to contribute to smoking uptake and continued use among younger generations of Southeast Asian youth. Such programs should build upon the positive anti-tobacco norms that are emerging in these populations.

It is important to note that this research project served as an important catalyst for change in Minnesota’s Southeast Asian communities. This research project led community leaders to elevate tobacco use to the level of an important community priority that needed to be addressed. Some participants and members of the research team from the four ethnic groups became advocates for tobacco control. They also became key participants in subsequent research projects and community interventions aimed at shifting social norms related to tobacco use and motivating current tobacco users to quit. These projects, in which community members were active participants, included the development of tobacco control messages, the integration of community messages into major community activities, and the use of community health workers to address tobacco-related policies and social norms related to tobacco use at culturally-specific venues, including multiunit housing, places of worship, ethnic businesses and community events. These projects incorporated insights from this study including the need to involve family, community, and clan; the need to address smokers’ psychological stress and social isolation; and changing social rituals in order to eliminate the role of tobacco. Thus, this study demonstrates how formative research, utilizing principles of CBPR, examining cultural and historical shifts in tobacco use patterns in ethnic communities can inform the development of tobacco control strategies that will be more effective because they are culturally meaningful in taking account the dimensions of acculturation.

## Abbreviations

CBPR: Community-based participatory research
CIA: Central Intelligence Agency.
